# Artificial Teeth

**Published:** 1846-06

**Authors:** 


*Artificial Teeth.*—The manufacture and application of artificial teeth have been brought to such perfection during the last fifteen or twenty years, that, when properly fixed in the mouth, it is difficult for a common observer to tell wherein they differ from the natural organs. They are made so as not only to resemble these, but also to be worn with comfort and to subserve, to a considerable extent, the purposes for which they are designed. But notwithstanding the perfection to which this branch of the dental art has been brought, the bungling and clumsy manner in which they are constructed and applied by, perhaps, a majority of dentists, it is really questionable whether in the aggregate, they are not productive of more injury than benefit. That a vast amount of mischief does result from their application, is undeniable. But, however much this is to be regretted, it is an evil which cannot be remedied, as long as empiricism in dentistry is encouraged and prevails to the extent to which it at present does.

In the application of an artificial crown to the root of a natural tooth, the simplest of all the methods of inserting artificial teeth, much judgment is required to determine the circumstances under which the operation ought, or ought not to be performed. It may be productive of incalculable advantage or injury, as it may be performed under favorable or unfavorable circumstances. For example, an artificial crown skilfully applied to a perfectly sound root situated in a socket free from all disease and morbid tendency, and in the mouth of a person of a good constitution, constitutes the very best and most durable substitute that can be employed for the loss of the crown of a superior incisor or cuspidatus; but if applied to a dead root or to one situated in a diseased alveolus, or from which there is a discharge of fœtid matter, or to one which is very soft and situated in the mouth of an individual of an exceedingly susceptible habit of body, it may be productive of lasting and irreparable injury.

In the application of artificial teeth on plate, either with or without clasps, a much greater amount of judgment, skill, and a more perfect knowledge of the laws of the economy, both in health and disease, are required, in order to secure to the patient the advantages capable of being derived from such substitutes, and to prevent the injury liable to be produced by them when constructed and fixed in the mouth in an improper manner. It is important that they should be so adjusted and applied as to restore the symmetry and contour of the face and to be useful in the mastication of food, in enunciation, and to be worn with ease and comfort by the patient. To secure these advantages, the teeth should be of the right size and color, correctly arranged on the plate, and this latter accurately adopted to all the inequalities of the parts on which it is to rest. If the contrivance is to be held in the mouth by means of clasps, these should be wide, and so applied to the teeth intended to be embraced by them, as to give to it the greatest stability, and, at the same time, without exerting any unequal or undue pressure upon them, and in such a way that the piece may be easily and readily removed and replaced by the patient.

But preparatory to their application, the remaining teeth, if there be any in the mouth, and the gums should be in a healthy condition, and it is oftentimes necessary to wait from six weeks to six months, after having instituted the necessary preparatory surgical treatment, before artificial teeth, fixed on plate, can be permanently and advantageously applied, for the reason, that the shape and size of the alveolar border, after the removal of the teeth, undergo a very great and striking change. If the teeth be applied before the completion of these, the plate will soon lose its adaptation, and bear unequally on the parts which it covers, and thus, be productive of irritation, as well as afford lodgments in the places where it does not touch, to extraneous matter, which soon becomes offensive and imparts to the breath an offensive odor.

It is equally improper to apply a plate over the roots of teeth. Whenever it is done, the softer parts around them become inflamed and spongy, and vitiate the secretions of the mouth. In this way, they are not only rendered hurtful to the remaining teeth, but also unfit for the purpose for which they are designed. Besides, a dental substitute thus applied, cannot be worn with comfort or satisfaction, nor be made to subserve any valuable purpose whatever.

But it is often necessary to apply artificial teeth almost immediately after removing the natural ones. In this case, a temporary set should be prepared, which, however, should only be worn when it is absolutely necessary, but in no case should a plate be applied over a dead root.

When the substitute is held in the mouth by means of clasps, it should be removed twice a day, and cleansed. The teeth, too, to which the clasps are applied, should also be thoroughly cleansed every time it is removed. These are pre cautions which should be strictly observed by all who wear artificial teeth inserted in this manner. If they be neglected, the secretions of the mouth which get between the clasps and the teeth, will become corrosive, and, sooner or later, cause the destruction of the latter.

The foregoing remarks are thrown out as hints to the young and inexperienced practitioner. We might say much more, but our limits, at present, will not permit us to enlarge upon the subject. At some future period we may enter more into detail.—*Stockton's Dental Intelligencer.*

